# A machine learning-based comparative analysis of surrogate models for design optimisation in computational fluid dynamics

**DOI:** 10.1016/j.heliyon.2023.e18674

**Published:** 2023-07-26

**Authors:** Azfarizal Mukhtar, Ahmad Shah Hizam Md Yasir, Mohamad Fariz Mohamed Nasir

**Affiliations:** a Institute of Sustainable Energy, Putrajaya Campus, Universiti Tenaga Nasional, Jalan IKRAM-UNITEN, 43000 Kajang, Malaysia; b College of Engineering, Putrajaya Campus, Universiti Tenaga Nasional, Jalan IKRAM-UNITEN, 43000 Kajang, Malaysia; c Faculty of Resilince, Rabdan Academy, 65, Al Inshirah, Al Sa'adah, 22401, PO Box: 114646, Abu Dhabi, United Arab Emirates; d STARE Resources Sdn. Bhd., Wisma Rampai, 2-4-29, Fourth Floor, Jalan 34/26, Taman Sri Rampai 53300 Wilayah Persekutuan Kuala Lumpur, Malaysia

**Keywords:** Design of experiment (DOE), Polynomial regression (PR), Kriging-based model, -Surrogate model, Underground shelter

## Abstract

Complex computer codes are frequently used in engineering to generate outputs based on inputs, which can make it difficult for designers to understand the relationship between inputs and outputs and to determine the best input values. One solution to this issue is to use design of experiments (DOE) in combination with surrogate models. However, there is a lack of guidance on how to select the appropriate model for a given data set. This study compares two surrogate modelling techniques, polynomial regression (PR) and kriging-based models, and analyses critical issues in design optimisation, such as DOE selection, design sensitivity, and model adequacy. The study concludes that PR is more efficient for model generation, while kriging-based models are better for assessing max-min search results due to their ability to predict a broader range of objective values. The number and location of design points can affect the performance of the model, and the error of kriging-based models is lower than that of PR. Furthermore, design sensitivity information is important for improving surrogate model efficiency, and PR is better suited to determining the design variable with the greatest impact on response. The findings of this study will be valuable to engineering simulation practitioners and researchers by providing insight into the selection of appropriate surrogate models. All in all, the study demonstrates surrogate modelling techniques can be used to solve complex engineering problems effectively.

## 1 Introduction

Engineering optimisation deals with the search for an optimal design of a system or element. The use of design optimisation techniques in engineering has been growing rapidly. In spite of the steady advance in computing architecture, the computational cost needed for carrying out a great number of simulations (due to many design variables) is still a major concern amongst the design engineers. Moreover, this approach often addresses one design at a time, whereby a designer might not be able to make a good design decision as they might fail to discover the functional relationship between the design variables and the responses [1]. In order to circumvent this problem, approximation model or surrogate model [2] can be adopted. There are numerous surrogate models available nowadays. Amongst these models, the Polynomial Regression (PR) [[3], [4], [5]] and the Kriging-based models [[6], [7], [8], [9]] are the popular ones in engineering [10,11]. More information regarding the application of surrogate models in engineering optimisation can be found in Refs. [1,10,12].

Recently, the number of studies focusing on applying different surrogate models (e.g., PR and Kriging-based) in optimisation has increased. However, it seems that the choice of surrogate model is very problem-dependent, and even for the same problem, small variations in the DOE can change the ranking of the methods [13,14]. Some comparative studies on PR and Kriging-based models in design optimisation have been reported [15,16]. Their modelling accuracies have been compared as well [17]. On the other hand [18], studied the advantages and disadvantages of surrogate models such as PR, Kriging, Multivariate Adaptive Regression Splines (MARS), and Radial Basis Function (RBF). The design sensitivities of PR and Kriging-based models have been reported, whereby these surrogate models were enhanced and the best strategy for model training was presented [19]. Also, the error measurements for PR and Kriging-based models in terms of noise-free functions have been investigated [11]. Some detailed reviews of the literature mentioned above are tabulated in Table 1 to provide some useful insights of design optimisation using various surrogate models. Despite the availability of numerous surrogate models, the choice of the appropriate model for a given dataset is still lacking, which raises concerns among design engineers.

**Table 1:** An overview of the comparative studies of different surrogate models with indication of the test problem, experimental design, model choice, and design variables.

Authors (year)	Test Problem	Experimental Design	Model Choice	Design Variables
Goel et al. [11]	Cantilever beam, Branin-Hoo function, Camelback	LHDs	PR, Kriging	2-6 variables
Simpson et al. [15]	Aerospike nozzle problem	OA	PR, Kriging	3 variables
Ahmed & Qin [16]	Hypersonic spiked blunt bodies	LHDs	PR, Exponential Kriging, Gaussian Kriging, General Exponential Kriging	3 variables
Giunta & Watson [17]	High-Speed Civil Transport Aircraft	D-optimal	PR, Kriging	5 and 10 variables
Jin et al. [18]	13's Mathematical problem, Vehicle handling	LHDs	PR, MARS, RBF, Kriging	2-16 variables
Rijpkema et al. [19]	Finite Elements Models	Full Factorial, LHDs	PR, Kriging	3 variables
Devanathan & Koch [20]	Branin function, Michalewicz function, Ackley path function,	Full Factorial, OSFD, Random	PR, Kriging, Blind Kriging, RBF, EBF, Chebyshev	random

LHDs = Latin Hypercube Sampling, PR = Polynomial Regression, OA = Orthogonal Array, MARS = Multivariate Adaptive Regression Splines, RBF = Radial Basis Function, OSFD = Optimal Space Filling Design, EBF = Elliptical Basic Function.

The choice of DOE, the sensitivity of the parameters to the response, and the suitability of the fitted surrogate model are a few crucial issues that must be addressed when using surrogate models for design optimisation. Barton [21] argues that addressing these concerns is necessary for ensuring the quality and dependability of the surrogate model. In this study, we aim to address the difficulty of understanding the relationship between inputs and outputs of complex computer codes by optimising a computational fluid dynamics (CFD) model of an underground shelter using PR and kriging-based models [22]. Their accuracy, sensitivity of design variables to response, model adequacy, and efficiency have been reported. In addition, the current results could guide the selection of an appropriate model for a given data set and demonstrate how surrogate modelling can be utilised to solve complex engineering problems.

The study focuses primarily on comparing the performance of PR and Kriging-based models as surrogate modelling approaches, rather than considering other popular methods such as Support Vector Regression (SVR) [23], 10.13039/100014230Gaussian Process Regression (GPR) [23], Radial Basis Function (10.13039/100004072RBF) [24] and Artificial Neural Networks (10.13039/100011554ANN) [25,26]. The decision to exclude these alternative methods can be attributed to several factors. First, the choice of modelling techniques in a research study is usually determined by the context in which the study is conducted. PR and Kriging-based models [4,5,8,27,28] were chosen because of their suitability to the problem under study and their widespread use in the field. Secondly, by focusing exclusively on PR and Kriging-based models, the study is able to conduct a more detailed and focused comparative analysis and explore in depth the strengths, weaknesses and design optimisation performance of these two methods. Finally, it is important to note that the exclusion of other methods does not diminish their value or relevance. Different surrogate modelling techniques have their own advantages and may be more suitable for different scenarios.

Overall, it is obvious that further comprehensive studies of surrogate models are needed to establish clear selection criteria for different datasets. Such studies can help overcome current limitations and ensure that surrogate models are widely used in design optimisation.

## 2 Related work

Surrogate modelling techniques [[29], [30], [31], [32], [33]] have gained popularity in optimising engineering design problems, with researchers focusing on different applications. Koziel and Pietrenko-Dabrowska [34,35] contribute to this field by addressing the challenges of designing and optimising high-frequency systems, especially antennas and microwave circuits. They emphasise the importance of incorporating automation and optimisation techniques into the design to achieve optimal performance while addressing conflicting objectives such as electrical properties, physical size and cost. To overcome the computational cost of full-wave electromagnetic analysis, the authors propose the use of surrogate modelling techniques.

Koziel and Pietrenko-Dabrowska [36]also explore several methods to improve the effectiveness of surrogate modelling, including triangulation of reference designs, nested kriging and explicit dimensionality reduction. These techniques contribute to accurate and efficient surrogate models by encompassing the Pareto front with small regions of parameter space. The authors also focus on the application of surrogate modelling techniques to inverse problems in antenna design. They propose a framework for global optimisation of multiband antennas by combining metaheuristic techniques, surrogate modelling and sequential sampling methods. Their approach includes a knowledge-based inverse surrogate that accounts for essential response characteristics and enables cost-effective design optimisation.

Furthermore, Koziel and Pietrenko-Dabrowska [37] emphasise in their other work, the consideration of manufacturing tolerances and uncertainties in antenna design. They present an approach that uses surrogate modelling techniques for design centering of multiband antennas. By establishing a functional relationship between response characteristics and antenna geometry parameters through knowledge-based inverse regression models, the authors enable the prediction of parameter vectors that increase the probability of meeting design requirements under uncertainty. This approach significantly reduces the computational costs associated with the design centering process.

In addition to using surrogate models, researchers have explored the combination of machine learning (ML) [23,[38], [39], [40], [41], [42], [43]] genetic algorithms (GA) [23,38,[43], [44], [45], [46], [47]] design of experiments (DoE) [13,48] and computational fluid dynamics (CFD) [4,5,8,12,27,28,49] in engineering optimisation. Moiz et al. [43] presented an integrated ML and GA approach to optimising internal combustion engines, achieving comparable results to traditional CFD-GA approaches with significant time and cost savings. Badra et al. (2021) [39] developed a machine learning approach (ML-GGA) for optimising compression ignition petrol engines, which has higher accuracy and robustness compared to ML-GA. Badra et al. [40] conducted a comprehensive study to optimise the combustion system of a compression ignition engine running on commercial petrol and achieved significant improvements in engine performance and emissions with their approach ML-GGA. Owoyele et al. [50] proposed an automated surrogate-based optimisation approach called AutoML- GA for internal combustion engines that addresses the challenges of selecting optimal ML hyperparameters and unknown training data requirements.

In summary, the studies discussed highlight the benefits of using surrogate modelling, ML-GA and Auto ML-GA approaches to design optimisation. By incorporating advanced computational techniques such as surrogate modelling, ML, GA and CFD, these methods provide efficient and cost-effective solutions for optimising designs considering multiple objectives and uncertainties. They provide valuable insights and strategies for improving performance and reducing computational effort in engineering optimisation. However, this paper focuses specifically on the comparison of PR and kriging-based surrogate models in design optimisation, focusing on their strengths and weaknesses and the integration of CFD simulations.

## 3 Surrogate model and optimisation structure

Since the past few decades, the surrogate model approach has been adopted by many Heating Ventilation and Air-Conditioning (HVAC) practitioners. Each surrogate model is associated with numerical fitting. For instance, the PR method uses the Least Squares Curve Fit (LSCF) method while the Kriging-based method uses the Best Linear Unbiased Predictor (BLUP) method. The construction of PR or Kriging-based model depends on the location of design points (in a design space). This design space can be defined by the lower and upper bounds of the independent parameters, thereby forming a n-dimensional cube. More explanation on these surrogate models can be found in Ref. [1].

### 3.1 Polynomial regression (PR)

PR is known to be straightforward and computationally efficient. It can be easily constructed; however, it is less accurate compared to more advanced schemes such as the Kriging-based model. PR is classically adopted in physical experiments. Nowadays, PR is applied in numerical modelling as well. PR is a statistical modelling technique that uses regression analysis to create a polynomial approximation of the computer analysis code [4,5]. This regression formula can be expressed in any order; however, the first- and second-order forms are the most popular ones. A second-order polynomial (Eq. (1)) was employed in the current study:

(1) $$y=\beta_{o}+\sum_{i=1}^{k}\beta_{i}x_{i}+\sum \sum_{i<j}\beta_{ij}x_{i}x_{j}+\sum_{i=1}^{k}\beta_{ii}x_{i}^{2}$$

Here, y is the predicted response value, β represents the unknown regression coefficients, and $x_{i}$ and $x_{j}$ refer to the design variables. Using the conventional least squares equation, the polynomial coefficient β can be estimated by minimising the sum of squared deviations between the predicted response value y(x) and the actual response value y(x). The equation can be expressed as follows (Eq. (2)).

(2) $$\beta ={\left[X^{T}X\right]}^{-1}X^{T}y$$

In the situation where X has linear independence, the existence of the ${\left[X^{T}X\right]}^{-1}$ is then guaranteed. The vector y denotes the set of observed responses across all design points. Therefore, the following representation of the matrix X (Eq. (3)) can be described accordingly.

(3) $$X\;\left(n\times m\right)=\left[\begin{array}{ccc} 1 & x_{11} & \cdots \\ \vdots & \vdots & \cdots \\ 1 & x_{n1} & \cdots \end{array}\;\begin{array}{ccc} x_{11}x_{12} & \cdots & x_{1k}^{2}\\ \vdots & \ddots & \vdots \\ x_{n1}x_{n2} & \cdots & x_{nk}^{2} \end{array}\right]$$

In this context, m, which is defined as (k+1)(k+2)/2, stands as a representative modelling term within the quadratic polynomial. The evaluation of the predicted response values at a new point $\bar{x}$ can be efficiently achieved by replacing the respective $\bar{x}$ within the polynomial equation.

### 3.2 Kriging-based model

Kriging is also known as a non-parametric interpolation method for Design and Analysis of Computer Experiment (DACE) [6,7]. In contrast to PR, Kriging was initially adopted for computer experiments via deterministic errors. The basic equation for the Kriging-based model (Eq. (4)) can be written as:

(4) y(x)=f(x)+Z(x)

Here, f(x) is a low-order polynomial that interpolates the design points. However, as reported by Sacks [7], the term f(x) in Eq. (4) is typically taken as a constant value for modelling complex input-output relations in which the output can be viewed as β. The equation can be expressed as follows (Eq. (5)).:

(5) y(x)=β+Z(x)

Here, β is an unknown constant to be estimated from the observed response value, while Z(x) is a realization of the normal distributed Gaussian stochastic function assumed to have zero mean, spatial covariance of the process variance $\sigma^{2}$ and the correlation matrix function R. The covariance matrix of Z(x) (Eq. (6)) can be written as:

(6) $$cov\;\left(Z\right)=\sigma^{2}\;R\left(x^{i},x^{j}\right)$$

Here, $R\left(x^{i},x^{j}\right)$ is the symmetric correlation matrix with values of unity along the diagonal. Then, the correlation function R is given as an exponential correlation function [6] written as (Eq. (7)):

(7) $$R\left(x^{i},x^{j}\right)=\exp\left[-\sum_{l=1}^{k}{\theta_{l}\left|x_{l}^{i}-x_{l}^{j}\right|}^{2}\right]$$

Here, i and j refer to two design points, l refers to the design parameter, k refers to the number of design parameters, and θ refers to the unknown correlation parameters, which can be estimated from the Maximum Likelihood Estimation (MLE) as described by Booker [6]. Then, the unknown constant β and the process variance $\sigma^{2}$ can be calculated using the generalized least squares. The equation can be expressed as Eqs. (8), (9):

(8) $$\beta ={\left[X^{T}R^{-1}X\right]}^{-1}X^{T}R^{-1}y$$

and

(9) $$\sigma^{2}=\frac{1}{n}{\left(y-X\beta \right)}^{T}R^{-1}\left(y-X\beta \right)$$

Here, X is an n×m matrix of design points depending on the choice of f(x). However, the results of β and $\sigma^{2}$ depend on the unknown correlation parameters θ. Once the design parameters and correlation function are estimated from the best linear unbiased prediction method, the values of the predicted response at a new point $\bar{x}$ can be computed from Eq. (10):

(10) $$\hat{y}\;\left(\bar{x}\right)=\beta +r^{T}\left(\bar{x}\right)R^{-1}\left(y-X\beta \right)$$

where $r^{T}\left(\bar{x}\right)$ is the correlation vector between $\bar{x}$ and all design points.

### 3.3 Screening optimisation approach

The optimisation sequence is the ultimate aim of performing a surrogate model analysis in engineering problems. The two surrogate modelling techniques used in the current study involved analyses of several responses. As such, the Shifted Hammersley Sampling [51] was used for optimising the surrogate model responses because this sampling method is computationally efficient. The conventional Hammersley Sampling algorithm is a quasi-random number generator. It has a very low discrepancy; therefore, it is suitable for quasi-Monte-Carlo simulations. As highlighted by Diwekar and Kalagnanam [51], the discrepancy is a quantitative measure for optimally approximating the sequence uniform distribution. In other words, due to the inherent properties of the Monte Carlo simulation, this sampling method provides an unbiased search space and better uniformity for the design space. Also, it does not have any effect on the dimensionality.

The Hammersley Sampling algorithm can be constructed using a radical inverse function. Any integer n can be written in a sequence radix-R format:

(11) $$n=n_{m}n_{m-1}\cdots n_{2}n_{1}n_{0}=n_{0}+n_{1}R+n_{2}R^{2}+\cdots +n_{m}R^{m}$$

Here, $m=\left[\log_{R}n\right]=\left[\ln\;n/\ln\;m\right]$ and the square brackets denote the integral part. Then, the radical inverse function is defined by revising the order of digits of Eq. (11) about the decimal point in Eq. (12):

(12) $$\Phi_{R}\left(n\right)=0.n_{0}n_{1}n_{2}\cdots n_{m}=n_{0}R^{-1}+n_{1}R^{-2}+\cdots n_{m}R^{-m-1}$$

Thus, the Hammersley points on the k-dimensional search space can be expressed as Eq. (13):

(13) $$z_{k}\left(n\right)=\left(\frac{n}{N},\Phi_{R_{1}}\left(n\right),\Phi_{R_{2}}\left(n\right),\cdots ,\Phi_{R_{k-1}}\left(n\right)\right)\;n=1,2,\cdots ,N$$

where $R_{1},R_{2},\cdots R_{k-1}$ are the first k-1 prime numbers and the Hammersley points are $x_{k}\left(n\right)=1-z_{k}\left(n\right)$.

## 4 Design of experiment (DOE)

### 4.1 Central Composite Design (CCD)

Central Composite Design (CCD) is the most popular experimental design method for fitting a PR model. It is known as one of the classical experimental design methods. It has a five-level fractional factorial designs, which is developed for fitting a second-order polynomial. CCD involves a two-level factorial points ($2^{k}$) augmented by two-star points and $n_{0}$ center points [1] defined on each factor. In addition, this method provides an alternative to experimental design for full factorial design (3^N^) while dealing with PR models. Technically, the method requires an experiment number of $N=2^{k}+2k+c_{p}$, where k is the number of variables and $c_{p}$ is the replicate number of the central point [1]. In contrast, CCD tends to consistently distribute the design points around the edge and center of the design space.

### 4.2 Box-Behnken Design (BBD)

Box-Behnken Design (BBD) is an independent three-level of second-order experimental design method in which it does not contain an embedded factorial design [5,52]. It is also one of the classic experimental designs originated from the theory of DOE, which is useful in building a second-order PR model for the response variable. The locations of the design points could be in the median of each design area. The BBD requires a smaller number of design points, increasing efficiency and cost-effectiveness compared to traditional experimental design methods. In contrast to CCD, the focus of BBD is primarily on the design points that lie at the boundaries of the design area. From a technical point of view, BBD requires several experiments denoted by $N=2k\left(k-1\right)+c_{p}$, where k is the number of variables and $c_{p}$ is the number of central point's [1].

### 4.3 Latin Hypercube Designs (LHDs)

Latin Hypercube Designs (LHDs) have been widely used in DACE, and it is also known as a stratified sampling technique. This method is an advanced form of the Monte Carlo Sampling which avoids clustering samples. In other words, the design does not replicate any row or column with other points. In addition, LHDs involve non-uniform distribution of points over the design space. This design is a complex statistical design, which can fit well with the Kriging-based model [53]. It has been found to be efficient in estimating the predicted response value [54]. Technically, it is targeted at the input space formed by the k-dimensional cube in n runs for the k factors ($0\leq d_{i:j}\leq 1\;with\;i=1,\cdots ,n\;and\;j=1,\cdots ,k)\text{.}$ More details on LHDs can be found in Refs. [[53], [54], [55]].

### 4.4 Optimal Space Filling Design (OSFD)

Optimal Space Filling Design (OSFD) is the extended form of LHDs for DACE. It is also known as the Optimal Latin Hypercube [54]. In general, the LHD approach is optimised through several iterations. A more uniform space distribution of design points can be achieved via maximizing the distance between points (without point-sharing in rows or columns) [54,56]. This method is effective for complex surrogate model techniques such as Kriging-based model and Neural Network [48]. It seems that the coverage of OSFD is better than that of LHDs when dealing with a more substantial number of design points. Hence, it is good in modelling the actual behaviour of response influenced by many design variables.

## 5 Case study description

The current case study deals with the optimal design of ventilation shafts for ventilating an underground shelter [49]. The ventilation shafts consist of inlet shaft, outlet shaft and elbow shaft. Basically, the ventilation shaft is widely used in channelling fresh air into the shelter. As argued by Shetabivash [57] and Etheridge [58], the size of the opening has a significant influence on the airflow pattern inside the building. Edward and Randall [59] reported that inlet and outlet ventilation shafts of equal opening size would produce the highest ventilation rate per unit area. A similar argument was also presented by Andersen [60], in which the author found that the ventilation rate was dependent on the inlet or outlet areas as well as the vertical opening distance due to the buoyancy effect. Thus, the relationship between the ventilation rate and the opening areas must be considered when designing a ventilation system for an underground shelter.

### 5.1 CFD configuration, model geometry and mesh generation

**Fig. 1:**
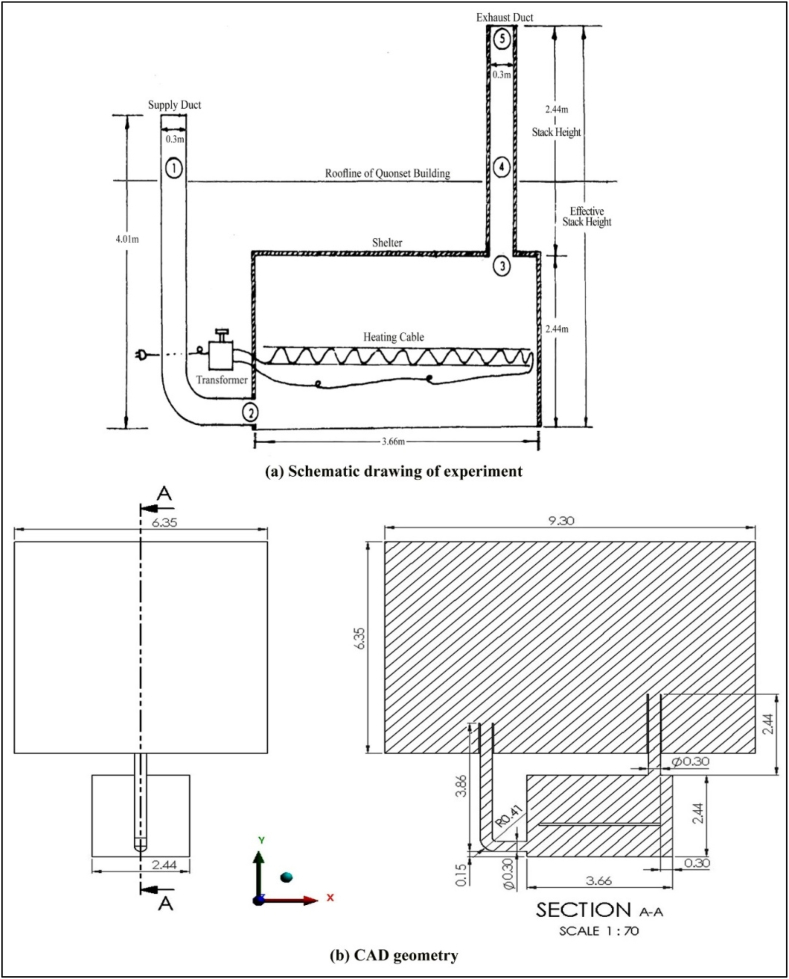
Schematic drawing of the underground shelter in this study.

**Fig. 2:**
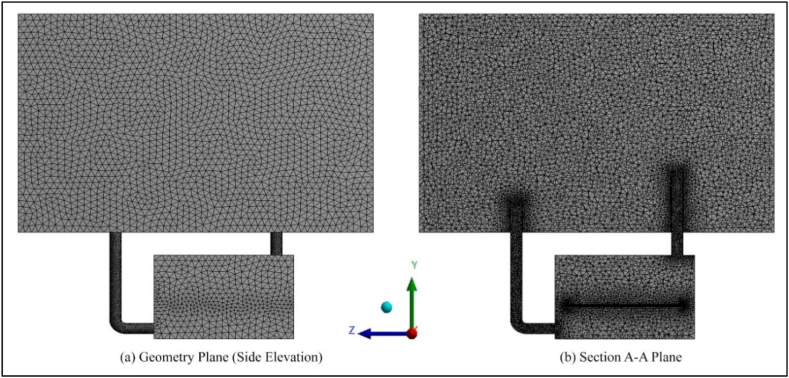
Computational surface grid of the simulation model.

In this study, the commercial software Computational Fluid Dynamics (CFD) based on the finite volume principle (namely ANSYS Fluent) was used to solve the Reynolds Averaged Navier-Stokes (RANS) equations. These equations describe the turbulent flow dynamics in the shelter. The experimental results [22] were used to validate the current CFD model. This shelter is a single-occupancy emergency shelter. A three-dimensional computer-aided design (CAD) of the experimental model was created specifically for the simulation (see Fig. 1). As depicted in Fig. 1b, the upper rectangular area was used to replicate the atmospheric circumstances of the actual experiment. The use of this condition allows the induction of air circulation through the inlet duct entrance, mimicking the authentic experimental environment. The heating cable was designed to replicate the heat release of a person in an enclosed space. Detailed description of the test case can be found in Ref. [28]. An unstructured tetrahedral mesh was then used to discretize the model (see Fig. 2a). Finer meshes were used in critical areas such as the ventilation shaft and the heating cable (see Fig. 2b). Six models with different element sizes were developed and simulated to investigate the effects of mesh size on flow results. The results are discussed in 4.3.

### 5.2 Computational technique and boundary conditions

The flow was considered stationary and incompressible. The realizable k−ε model was used to represent the flow turbulence as it can accurately reproduce the characteristics of the flow in an indoor setting [58]. The pressure-velocity coupling algorithm, i.e. SIMPLEC, was used. Furthermore, the use of higher order convective schemes is required to ensure accurate flow [8,28]. To ensure both accuracy and stability, all governing equations were discretised with second-order upwind schemes. The governing equations such as mass (14), momentum (15) and energy (16) conservation equations are:

(14) $$\frac{\partial \rho }{\partial t}+\nabla .\rho \vec{v}=0$$

(15) $$\frac{\partial }{\partial t}\left(\rho \vec{v}\right)+\nabla ∙\left(\rho {\vec{v}}^{2}\right)=-\nabla p+\nabla ∙\tau +\rho \beta \vec{g}\left(T-T_{0}\right)$$

(16) $$\frac{\partial }{\partial t}\left(\rho E\right)+\nabla ∙\left(\vec{v}\left(\rho E+p\right)\right)=-\nabla ∙\left(k\nabla T\right)+\Phi +S_{h}$$

In this context, ρ signifies density, t denotes time, $\vec{v}$ is the velocity vector, p corresponds to pressure, τ is the viscous stress tensor, $\vec{g}$ refers to gravitational acceleration, E encapsulates total energy, k is indicative of the thermal conductivity of the fluid, T is the absolute temperature, $S_{h}$ represents the source term and Φ refers to viscous dissipation. The term $\rho \beta \vec{g}\left(T-T_{0}\right)$ on the right-hand side (RHS) of Eq. (15) symbolises the buoyancy force, where β is the thermal expansion coefficient, $\rho_{0}$ is the reference density of the flow and $T_{0}$ is the operating temperature. The integration of the buoyancy model for the simulation of indoor air flows is documented in Ref. [61].

The simulation was then run for about 850 iterations to obtain a solution that showed convergence. The solution was considered to have converged when there were no longer any significant deviations in variables such as velocity, energy and turbulence (the scaled residual errors RMS dropped to 10^−4^) and the area had a net imbalance of less than 1%. To speed up the convergence of the solution, the Algebraic Multi-Grid (AMG) technique was used. The aggregation of the mass flow rate at the outlet of the opening shaft allowed the calculation of the post-simulation ventilation rate.

**Fig. 3:**
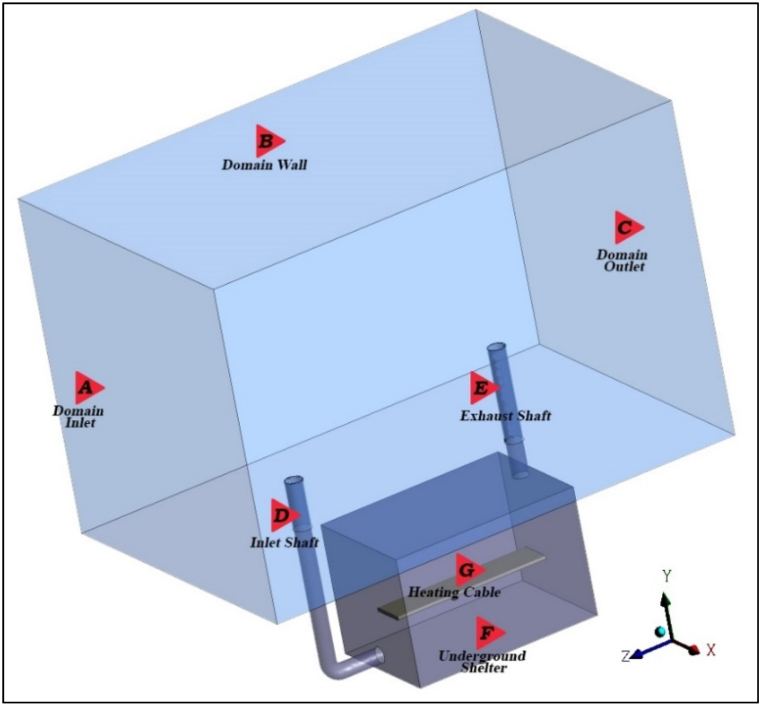
Computational domain of boundary conditions.

The boundary conditions were configured to match the experimental conditions as described in Ref. [22]. An invariant wind profile was specified at the domain inlet, while a zero static pressure was determined at the domain outlet. A free-slip condition was used for the domain wall (under atmospheric conditions). The surfaces of the ventilation shafts were treated as adiabatic, no-slip walls. The heat flux emanating from the heating cable was set to a value of 70 W/m^2^ [62,63]. The computational domain is shown in Fig. 3 and the boundary conditions are summarised in Table 2.

**Table 2:** Boundary conditions for the simulation.

Domain Inlet	constant velocity, 2.68 m/s at a constant temperature, 23°C
Domain Outlet	atmospheric pressure
Domain Wall	free slip wall at a constant temperature, 23°C
Inlet Shaft	adiabatic, no-slip walls ($u_{i}=0$)
Outlet Shaft	adiabatic, no-slip walls ($u_{i}=0$)
Heating Cable	heat flux, 70 W/m^2^
Underground Shelter	adiabatic, no-slip walls ($u_{i}=0$)

### 5.3 Grid independent test and CFD validation

**Fig. 4:**
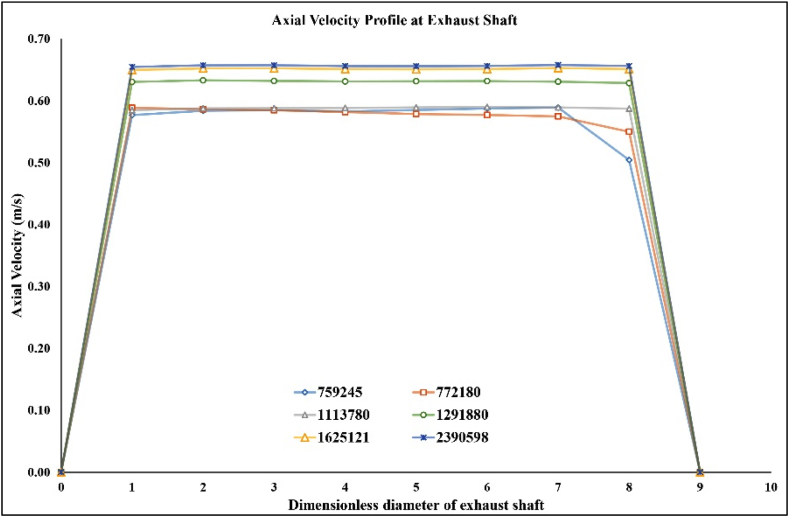
Verification charts.

Before the optimisation process of the Computational Fluid Dynamics (CFD) model, it is essential to compare the results of CFD with previous experimental results [22]. The Grid Independence Test (GIT) was first performed with six different grids (refer to Table 3). The range of ventilation rate deviation was between 3.35% and 14.05%, as shown in Table 3. The results generated with grids E and F do not seem to show any significant deviation. A corresponding result can be seen in Fig. 4, where the cases with larger mesh sizes (grids E and F) show analogous values for the axial velocity at the outlet of the opening shaft. Thus, when the number of cells reaches 1.63 million, the solution can be considered grid independent. The model with grid E was then selected for further flow analysis.

**Table 3:** Grid parameters for six different mesh sizes.

Grid	A	B	C	D	E	F
Element Size	Default	0.5	0.2	0.175	0.15	0.125
Number of Cells	759,245	772,180	1,113,780	1,291,880	1,625,121	2,390,589
ε	11.95%	14.05%	13.00%	6.5%	3.56%	3.35%

A comprehensive validation of the model CFD shown in Fig. 1 was carried out. The calculated ventilation rate was 0.0456 m^3^/s, in contrast to the measured ventilation rate of 0.0477 m^3^/s [22]. The percentage discrepancy between these two values was only 4.4%. Consequently, the current CFD model can be trusted to accurately predict the ventilation efficiency of naturally ventilated underground shelters. Detailed validation studies for the current model CFD can be found in Ref. [28].

## 6 Surrogate model methodology

**Fig. 5:**
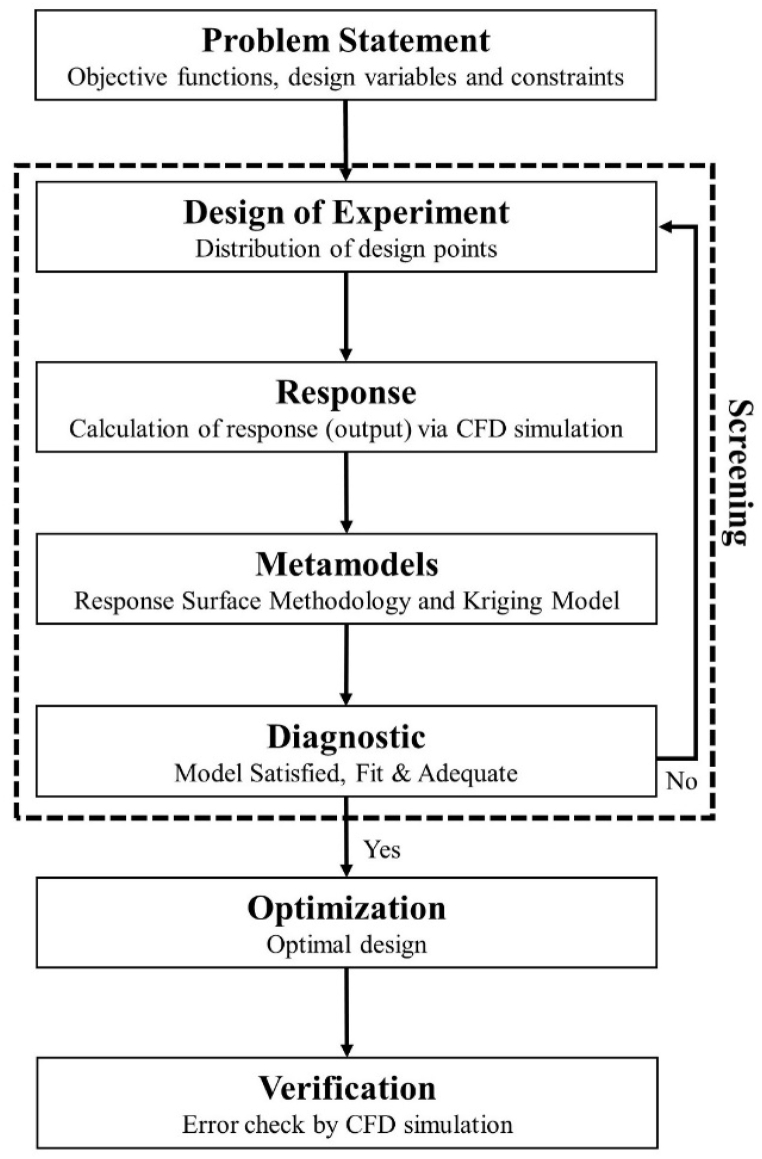
Flowchart of the optimisation process.

**Fig. 6:**
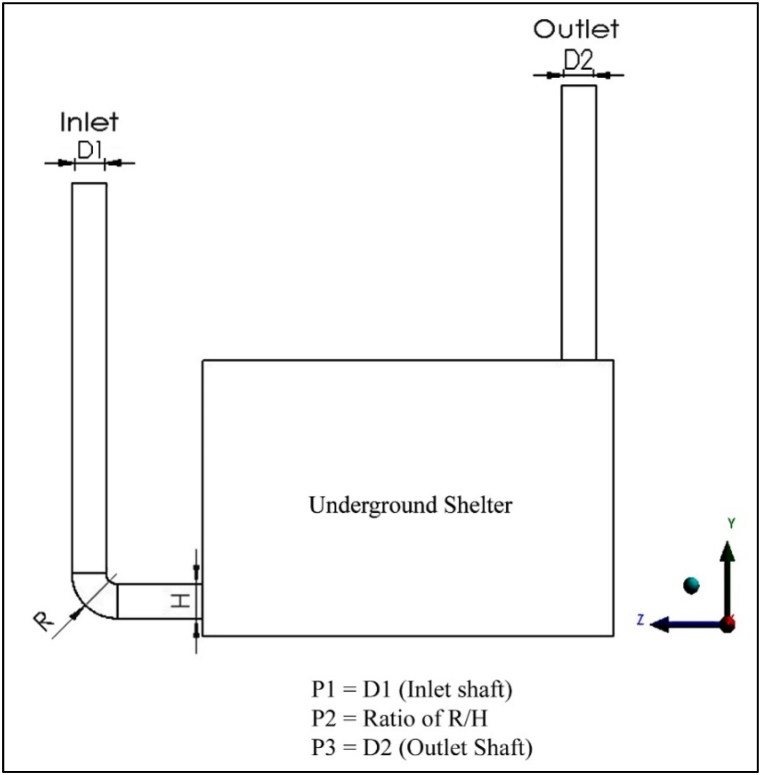
Design variables.

The model can be optimised once the CFD model is validated. Here, the ANSYS Design Exploration 16.0 software was used to solve the selected DOE and surrogate models [13]. Fig. 5 shows the optimisation process in this study. The first step of parametric and optimisation analysis consists of identifying the objective functions, design variables (input parameters), and constraints. In this study, the objective function is to maximize the ventilation rate. The selection of design variables is influenced by the findings of previous studies (presented in Section 4). The inlet opening (P1), the ratio of radius to height of inlet shaft (P2), and the outlet opening (P3) were expected to have a significant influence on the ventilation rate (response) (P4) required for one person inside the underground shelter (see Fig. 6). The ranges of the design variables (i.e. lower bound, upper bound and constraint) were reported in Ref. [22]. The design variables including their ranges are summarised in Table 4.

**Table 4:** Types of design variables with upper and lower boundary parameters.

Parameters		Name	Upper Bound (m)	Lower Bound (m)	Constraints
Factors (IV)	P1	Inlet Opening	0.1524	0.1016	–
	P2	Ratio of Elbow Shaft	2.50	0.75	≥ 0.75
	P3	Outlet Opening	0.1524	0.1016	–
Response (DV)	P4	Ventilation Rate (m^3^/s)			

The selection of a proper DOE is significant [13,14] while performing numerical experiments. In this study, the distribution of design points was determined by using four types of DOE, namely BBD, CCD, LHDs and OSFD. These types of DOE have been recommended for PR and Kriging-based models [3,48,53]. In this study, a DOE approach was used to efficiently locate the data points and improve the computational time and accuracy of the surrogate model. For the DOE analyses conducted in this study, a total of 14–15 data points were generated, each representing varying input parameters for the Computational Fluid Dynamics (CFD) model. In the current work, the accuracy of response prediction was expressed using the coefficient of determination *R*^*2*^ (i.e. Adjusted *R*^*2*^ and Predicted *R*^*2*^). In addition, the early prediction of the maximum and minimum search of ventilation rate was also investigated.

In this study, both PR and Kriging-based models were adopted for the purposes of regression and interpolation analyses, respectively. In this stage, the relationship between the input parameters and the response was investigated by examining the local sensitivity curves [64]. Local or global sensitivity analyses depend on the objectives of the study, the resources and the complexity of the model. Both methods have their advantages, but local sensitivity analysis is more appropriate for this study. Complex models make global sensitivity analysis computationally difficult and time-consuming [14,65] Local sensitivity analysis, on the other hand, focuses on a specific region of parameter space or a collection of variables, making it computationally more efficient [39,66]. This localised technique is useful for answering specific hypotheses that require a deep understanding of the model's behaviour in a particular domain. It allows targeted studies of how minor changes in input parameters affect model results in that region [8]. Local sensitivity analysis also helps prioritise factors by validating or quantifying their influence. When computational resources or time are limited, this strategy reduces the scope of the analysis and saves work. Also, in this study contour plot was used to visualize the response of the ventilation rate due to the input parameters. The plot outlines the minimum and maximum range of the response data. In fact, it also gives an early prediction of the possibility of the highest ventilation rate before the optimisation result is finalised. Table 5 presents the surrogate model techniques employed in this study.

**Table 5:** Surrogate model techniques.

Experimental Design	Model Choice	Model Fitting	Sample Approximation Technique
CCD	Second Order Polynomial	Least square regression	PR
BBD			
LHDs	Realization of a stochastic process	Best linear unbiased predictor (BLUP)	Kriging
OSFD			

The final step involves the selection of objective function and screening optimisation. As mentioned earlier, the objective function involves the maximization of ventilation rate. The sample set can be generated by screening in order to determine the effect of input parameters on the output parameters. Technically, there are three optimisation methods available in ANSYS Design Explorer, i.e. Shifted Hammersly Sampling (SHS), Multi-objective Genetic Algorithm (MOGA) and Non-linear Programming by Quadratic Lagrangian (NLPQL). SHS is a non-iterative direct sampling method using a quasi-random number generator based on the Hammersley algorithm which is good for obtaining the preliminary optimal solution. MOGA is a more advanced approach which is usually employed when there are multiple objective functions. NLPQL is a fast gradient-based local optimisation algorithm for single objective function. In the current study, we have decided to use SHS method which is a simple approach based on sampling and sorting. In fact, it supports multiple objective functions and constraints as well as all types of input parameter [67]. Moreover, this approach is able to provide a global overview of the design space and to identify the local minima.

## 7 Result and discussion

### 7.1 Sensitivity analysis

**Fig. 7:**
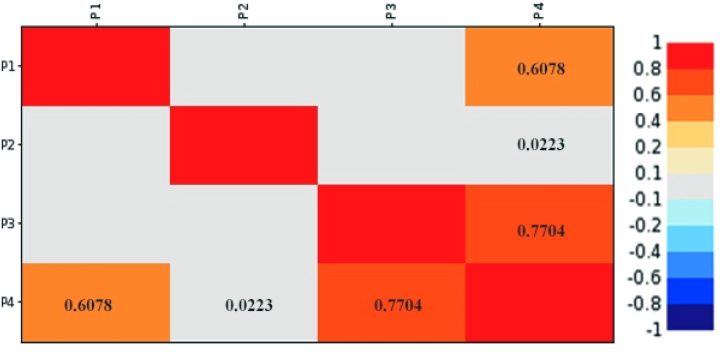
Correlation matrix.

**Fig. 8:**
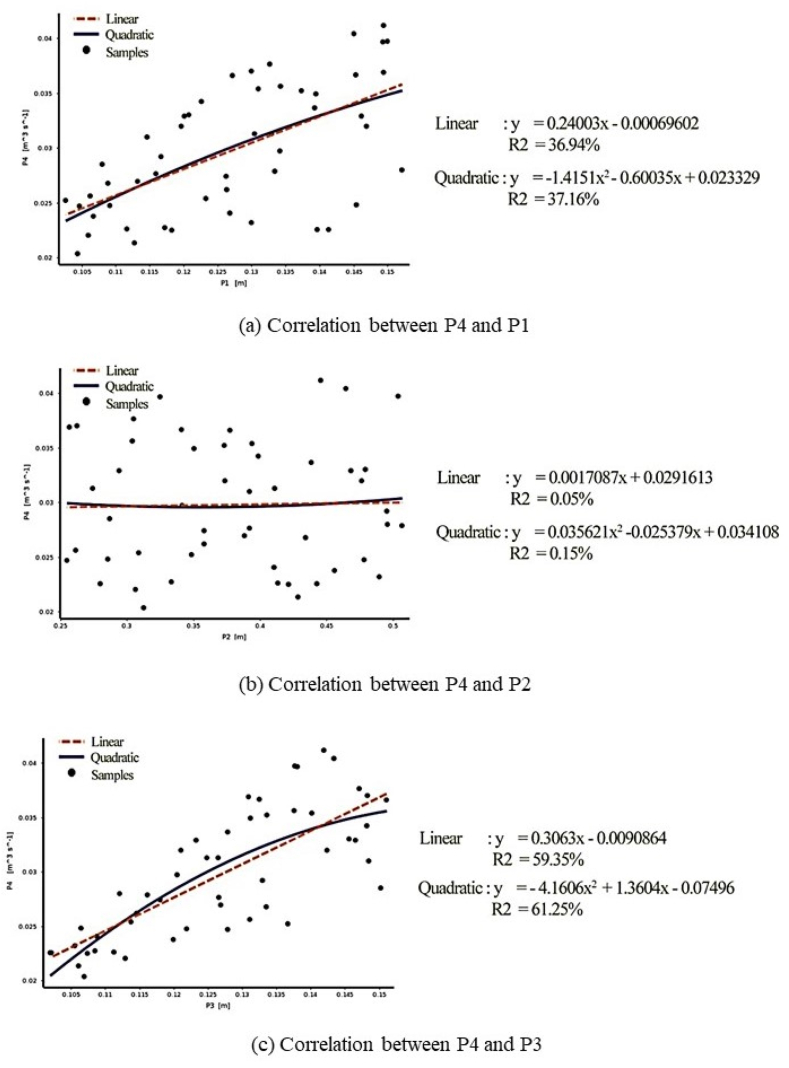
Correlation charts.

A correlation matrix was created to observe the response and its correlation with other parameters (factors). In other words, the strong and weak correlations of each factor with the design response should be determined. Pearson's rank correlation was used for this purpose, which requires 100 samples with 5% of the corresponding threshold for filtering along the correlation value [8]. The correlation matrix, as shown in Fig. 7, provides a visual representation of the correlations, allowing the user to see which factors have the strongest relationship with the response variable. This information can be used to prioritise which factors to focus on for optimisation or further investigation. The colour coded matrix that resulted indicated the strength of the correlation between each factor and the response variable. When the value is closer to the absolute value of 1, a stronger relationship is expected. According to the matrix, P3 has the most influence on P4 with a correlation value of 0.7704, followed by P1 with a correlation value of 0.6078 and P2 with a correlation value of only 0.0223. A scatter plot in Fig. 8 was also created to produce both linear and quadratic trend lines for the most highly correlated factors. The quadratic trend line performed better for each factor in this case, indicating a non-linear relationship between the factors and the response variable. As shown in Fig. 8 the highest estimated coefficient of determination (*R*^*2*^), with a percentage of 61.25%, is found for the correlation between P3 and P4 (see Fig. 8c). The coefficient of determination (*R*^*2*^) for the correlation between P1 and P4 is only 37.16% (see Fig. 8a), while the correlation between P2 and P4 has a lower coefficient of determination (*R*^*2*^) of 0.15% (see Fig. 8b). Overall, P1 and P3 showed a stronger correlation with a higher coefficient of determination (*R*^*2*^) than P4. The correlation matrix and scatter plot information can be used to prioritise which factors to focus on for optimisation or further investigation.

### 7.2 Comparison between the PR and kriging-based models

The simulation results of the PR and Kriging-based models can be compared by plotting the predicted response values against the observed values. The final surrogate model using different DOE methods is shown in Table 6. Typically, a good DOE model for each surrogate model would exhibit high Adjusted *R*^*2*^ and Predicted *R*^*2*^ values. Technically, the adjusted *R*^*2*^ value is the modification of *R*^*2*^ (the ratio of the model sum of squares to the total sum of squares) for the number of description terms in the model while the predicted *R*^*2*^ is used to identify the prediction of the model response based on the new observations and to give the first insight into the sensitivity of the design solution. Also, in the current study, we have identified the minimum and maximum search outputs of the response to yield a better prediction for the improvement of optimisation.

**Table 6:** PR and Kriging surrogate model with the best quality of fit using different DOE methods.

Surrogate model	DOE	Design Point	Max-min Search		Adj *R*^*2*^	Pred *R*^*2*^	Quality of Fit
			Min Output (m^3^/s)	Max Output (m^3^/s)			
PR	BBD	14	0.0192	0.0468	0.996	0.998	
	CCD	15	0.0189	0.0466	0.978	0.983	
Surrogate model	DOE	Design Point	Max-min Search		RMSE		Quality of Fit
			Min Output (m^3^/s)	Max Output (m^3^/s)			
Kriging-based	LHDs	15	0.0179	0.0476	1.3 × 10^−9^		
	OSFD	15	0.0167	0.0471	1.5 × 10^−5^		

As shown in Table 6, he PR method with BBD generates a smaller number of design points, which may result in less accurate model predictions due to a lower sampling density of the design space. PR may thus be appropriate for simpler systems with lower computational costs. Furthermore, PR assumes that the data is free of noise, which may result in inaccurate predictions in the presence of data uncertainties. When evaluating the max-min search result, the Kriging approach produces 0.0167 m^3^/s and 0.0476 m^3^/s, respectively. The points developed in the design space show that the Kriging-based approach is superior to PR. This is because kriging-based models are better at modelling nonlinear relationships and handling noisy data, which can lead to more accurate predictions in the presence of complex system behaviour and data uncertainties. Kriging-based approaches also perform well in well-sampled design spaces, where a higher number of design points is typically required to achieve higher surrogate model accuracy. In addition, kriging-based methods provide uncertainty estimates for the predicted values, which can help in evaluating the reliability of the model predictions. In general, the higher the number of design points, the higher the accuracy of the surrogate model. It appears that both LHDs and OSFDs produce greater coverage of points in the design space compared to CCD and BBD. It should be noted, however, that this result is only a preliminary prediction for determining the maximum and minimum likelihood of the result prior to performing the optimisation.

As noted from Table 6 (see Quality of Fit), the PR technique employs the Least Square Regression to fit the simulation results while the Kriging-based technique interpolates the simulation results and fits the Maximum Likelihood Estimation (MLE) as well as the Unbiased Linear Predictor-criterion (ULP) on the results. On the other hand, the polynomial model can be evaluated by computing the *R*^*2*^ error. In general, higher *R*^*2*^ indicates better Quality of Fit and accuracy. For instance, the PR + BBD model is more desirable, and it has a stronger correlation with the Quality of Fit (e.g. *R*^*2*^ = 0.998). Basically, the Quality of Fit is a statistical significance of the surrogate model coefficients, which describes how well the observations points are fitted. This method is known as local surrogate model-based optimisation. Meanwhile, for evaluating the interpolation model, cross-validation and Root Mean Square Error (RMSE) can be employed to give a more accurate approximation over the design points. This argument was also discussed in Refs. [15,68] and the method was known as global surrogate model-based optimisation. Since the Kriging-based techniques use cross-validation and RMSE, the integrity of the function should not be dependent on the values of Adjusted *R*^*2*^ and Predicted *R*^*2*^ because the more the parameters involved in the system, the higher the value of the regression sum of squares which might lead to value greater than 1.0 [69]. Indeed, in current study, the values of Adjusted *R*^*2*^ and Predicted *R*^*2*^ were equal to 1.0, as ANSYS automatically truncates those values that are above 1.0 [67]. However, according to equation (17):

(17) $$RMSE=\sqrt{\frac{1}{n}\sum_{i=1}^{n}{\left(y_{i}-{\vec{y}}_{i}\right)}^{2}}$$

the current RMSE value varies from $1.3\times 10^{-9}\;to\;1.5\times 10^{-5}\text{,}$ indicating that the regression function is adequate in representing the model. In other words, the closer the value of RMSE is to zero, the better the quality of fit. In fact, the current error estimations were obtained based on bias error rather than noise (as the error is stemming from model inadequacy instead of noise). Thus, it seems that Kriging-based model is more accurate than PR model. More explanations on the comparison between PR and Kriging-based models in terms of error measurement of noisy response can be found in Ref. [11].

For the sensitivity and response point of the design solution, it seems that PR has better sensitivity factors and higher response points as compared to the Kriging-based approach (see response point). Table 7 shows the variation of output with respect to the input parameters. From the results, it seems that the effects of P1 and P3 on the ventilation rate are more apparent than that of the ratio of elbow shaft (P2). Higher P1 or P3 would increase the ventilation rate. On the other hand, an opposite trend is observed for P2. Essentially, the local sensitivity study was conducted to examine the weight of each parameter around the response point. Each curve represents the impact of an enabled input parameter towards the output. Thus, PR is the best method to estimate interaction and even quadratic effects of the shape of response surface as PR was initially developed to analyse the cause-effect relationship of a physical experiment [3]. On top of that, it seems that both PR and Kriging-based approach reflect an important contribution of P1 and P3 on P4 which both of it detect a stronger effect from P3 (higher slope of the curves for P3). These findings are in qualitative agreement with the correlation matrix and charts (see Fig. 7, Fig. 8).

**Table 7:** PR and Kriging surrogate model with the best sensitivity and response point using different DOE methods.

Surrogate model	DOE	Inlet	Outlet	Response Point	Local Sensitivity Curves
PR	BBD	0.326	0.537	(0.5, 0.0309)	
	CCD	0.397	0.509	(0.5, 0.0307)	
	DOE	Inlet	Outlet	Response Point	Local Sensitivity Curves
Kriging-based	LHDs	0.353	0.533	(0.5, 0.0300)	
	OSFD	0.359	0.519	(0.5, 0.0301)	

**Fig. 9:**
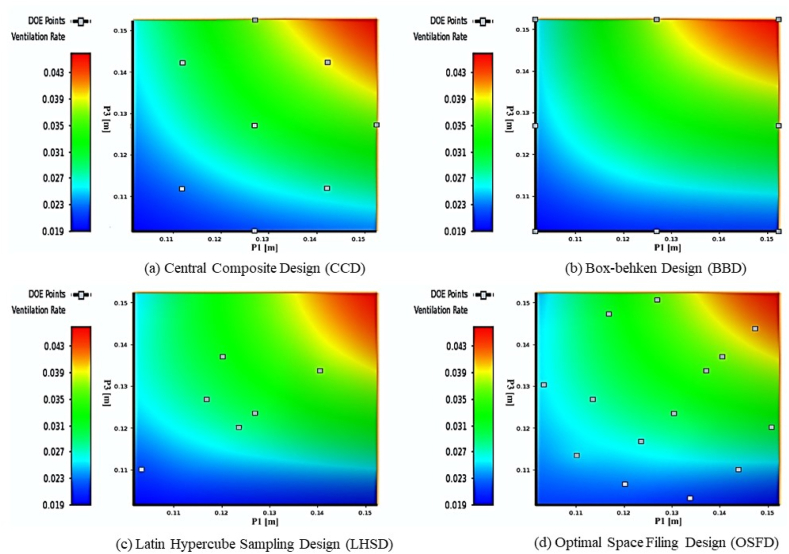
Contour plot of different DOE.

The Kriging-based approach, however, was developed to fix the random error produced by a computer experiment [7]. In addition to the sensitivity factors and response point, a graphical comparison between PR and Kriging-based approaches was conducted to visualize the highest ventilation rate predicted by all types of DOE. Fig. 9, presents the contour plots of P1, P3, and P4. All results are almost similar, where the highest ventilation rate can be identified at the top right corner of the contour plot between the P1 and P3 factors. As seen in Fig. 9, the *Z*-axis represents the predicted ventilation rates (P4) of PR and Kriging-based models, while the *X*- and *Y-* axes represent the strong correlation of factors (e.g. P1 is Inlet Opening, and P3 is Outlet Opening, respectively). Overall, the design point distribution depends on type of DOE. The DOEs used in the PR approach (e.g. CCD (see Fig. 9a) and BBD (see Fig. 9b)) tend to generate the design points near the boundary of a design space. On the other hand, the DOEs used in the Kriging-based approach (see Fig. 9c and d) are known to yield a more uniform distribution of design points over the design space [70]. Therefore, they are good in predicting a larger dispersion of objective values. This argument has been verified, as presented in Table 6, for which the Kriging-based approach can identify the highest and the lowest max-min search outputs of the ventilation rate due to the dispersed locations of the design points.

### 7.3 Screening optimisation results

The screening approach (Shifted Hammersley Sampling) was employed for each surrogate model. It is one of the optimisation methods used in the ANSYS Design Explorer. A total of 1000 distributed sample sets were generated to find the optimal design based on the objective function. The results of the screening method are summarised in Table 8. It can be seen that the predicted maximum ventilation rates using PR and Kriging-based models increase by 6.14% and 4.39% (relative to the initial design), respectively. To validate the optimised result, the predicted optimal design is modelled using CFD. Table 9 compares the values obtained from the Shifted Hammersley Sampling and CFD analysis. By examining the results, the maximum error is within 2%–4%, which is within the permissible range of the current problem.

**Table 8:** Summary of optimisation results.

Variables		Geometrical Dimensions			Ventilation Rate (m^3^/s)
		P1 (m)	P2 (m)	P3 (m)	
Initial Design		0.1524	1.67	0.1524	0.0456
PR	BBD	0.1524	2.13	0.1524	0.0484
	CCD	0.1524	2.13	0.1524	0.0484
Kriging-based	LHDs	0.1524	2.31	0.1524	0.0476
	OSFD	0.1524	2.31	0.1524	0.0470

**Table 9:** Summary of verification results.

Variables		Predicted Optimisation (m^3^/s)	CFD (m^3^/s)	Error (%)
PR	BBD	0.0484	0.0465	4.08%
	CCD	0.0484	0.0465	4.08%
Kriging-based	LHDs	0.0476	0.0461	3.25%
	OSFD	0.0470	0.0461	2.39%

## 8 Conclusion

This paper compared two common surrogate modelling techniques, namely PR and Kriging-based models, for optimising a CFD model by identifying the functional relationship between the design variables and the response. PR models use a polynomial function to fit the simulation results, while Kriging-based models use an interpolation technique to fit the simulation results. Both techniques can be used for optimisation, but their effectiveness depends on the specific problem and the nature of the design space.

PR was discovered to be computationally efficient and appropriate for model building because it requires fewer design points, resulting in less computation time. The Kriging-based model, on the other hand, is a more suitable method for evaluating the max-min search performance of the response. This is probably due to the large number of design points, which is beneficial for predicting a larger dispersion of the objective values. The number and location of the design points may also affect the performance of the model created. When examining the quality of fit, PR appears to rely on estimating the *R*^*2*^ error to capture the correlation between the predicted and observed points. In this case, the Kriging-based model is superior because all predicted and observed points are fitted with a more accurate function than the design points. Furthermore, we discovered that the kriging-based model has a lower error than the PR model. In fact, the efficiency of a surrogate model can be improved by including information such as design sensitivity. In this case, PR models are better suited for estimating interaction and quadratic effects of the input parameters, while Kriging-based models are better suited for identifying the main effects of the input parameters. SHS was used to predict the preliminary optimal solution for optimisation, and the optimised result of PR is slightly higher than that of the kriging-based model. The current evaluation of surrogate model performance is thought to be useful for the design of HVAC components.

All in all, there is no one-size-fits-all approach to surrogate modelling for optimisation. Both PR and kriging-based models have advantages and disadvantages, and the choice between them is determined by the nature of the problem and the design space. PR is computationally efficient and appropriate for model building, whereas kriging-based models are better for evaluating the response's max-min search performance. Kriging-based models are also better at fitting predicted and observed points with a more accurate function than the design points. The selection of an optimisation procedure should be based on a careful assessment of the performance of each procedure to determine which method provides the best results for optimisation.

## Author contribution statement

Azfarizal Mukhtar: Conceived and designed the experiments; Performed the experiments; Analyzed and interpreted the data; Contributed reagents, materials, analysis tools or data; Wrote the paper.

Ahmad Shah Hizam Md Yasir: Performed the experiments; Analyzed and interpreted the data.

Mohamad Fariz Mohamed Nasir: Contributed reagents, materials, analysis tools or data; Wrote the paper.

## Data availability statement

Data will be made available on request.

## Declaration of competing interest

The authors declare that they have no known competing financial interests or personal relationships that could have appeared to influence the work reported in this paper.
